# Characterization and extraction of the synaptic apposition surface for synaptic geometry analysis

**DOI:** 10.3389/fnana.2013.00020

**Published:** 2013-07-04

**Authors:** Juan Morales, Angel Rodríguez, José-Rodrigo Rodríguez, Javier DeFelipe, Angel Merchán-Pérez

**Affiliations:** ^1^Cajal Blue Brain Project, Facultad de Informática, Universidad Politécnica de MadridMadrid, Spain; ^2^Departamento de Arquitectura y Tecnología de Sistemas Informáticos, Facultad de Informática, Universidad Politécnica de MadridMadrid, Spain; ^3^Laboratorio Cajal de Circuitos Corticales, Centro de Tecnología Biomédica, Universidad Politécnica de Madrid and Instituto CajalCSIC, Madrid, Spain

**Keywords:** chemical synapses, three-dimensional, electron microscopy, active zone, postsynaptic density, surface extraction, data preprocessing, data visualization

## Abstract

Geometrical features of chemical synapses are relevant to their function. Two critical components of the synaptic junction are the active zone (AZ) and the postsynaptic density (PSD), as they are related to the probability of synaptic release and the number of postsynaptic receptors, respectively. Morphological studies of these structures are greatly facilitated by the use of recent electron microscopy techniques, such as combined focused ion beam milling and scanning electron microscopy (FIB/SEM), and software tools that permit reconstruction of large numbers of synapses in three dimensions. Since the AZ and the PSD are in close apposition and have a similar surface area, they can be represented by a single surface—the synaptic apposition surface (SAS). We have developed an efficient computational technique to automatically extract this surface from synaptic junctions that have previously been three-dimensionally reconstructed from actual tissue samples imaged by automated FIB/SEM. Given its relationship with the release probability and the number of postsynaptic receptors, the surface area of the SAS is a functionally relevant measure of the size of a synapse that can complement other geometrical features like the volume of the reconstructed synaptic junction, the equivalent ellipsoid size and the Feret's diameter.

## Introduction

Chemical synapses play a pivotal role in the exchange of information between neurons. They are formed by a presynaptic axon terminal and a postsynaptic membrane, separated by the synaptic cleft. At the presynaptic membrane, the active zone (AZ) contains the molecular machinery necessary for the rapid docking of synaptic vesicles and subsequent release of the neurotransmitter that they contain (Sigrist and Schmitz, 2011; Gundelfinger and Fejtova, 2012; Südhof, 2012). The size of the AZ is proportional to the number of docked synaptic vesicles (Schikorski and Stevens, 1997, 1999) and to the probability of vesicle release (Murthy et al., 2001; Branco et al., 2010; Matz et al., 2010; Holderith et al., 2012). The released neurotransmitter molecules interact with the postsynaptic receptors present in the postsynaptic density (PSD). The area of the PSD is proportional to the number of postsynaptic receptors (for AMPA receptors, for example, see Nusser et al., 1998; Kharazia and Weinberg, 1999; Takumi et al., 1999; Tarusawa et al., 2009). Therefore, the size and shape of the AZ and the PSD are of great interest in terms of synaptic function (e.g., Kubota and Kawaguchi, 2000; Arellano et al., 2007).

Both the AZ and the PSD are electron-dense structures that can be readily identified under the electron microscope. Although they are separated by the synaptic cleft, this space is not always visible, and the pre- and postsynaptic membranes may give the impression of being fused together when the synaptic junction is sectioned obliquely or parallel to the cleft (*en face*). In other words, the synaptic cleft is not clearly visible, and the AZ and the PSD cannot be resolved separately unless the plane of section is perpendicular to the synaptic junction (DeFelipe et al., 1999; Merchán-Pérez et al., 2009). This is an obvious limitation when trying to reconstruct the AZ and the PSD individually from serial sections, since only a small portion of synapses would be properly sectioned for this purpose. Two facts, however, can be exploited to extract a surface that is equivalent to both the AZ and the PSD, even if they appear as a single structure: (i) The AZ and the PSD are in close apposition, only separated by a narrow and uniform space, the synaptic cleft (Peters and Palay, 1996). (ii) Since the AZ and the PSD are located face to face, their surface areas are very similar (correlation coefficients over 0.97; see Schikorski and Stevens, 1997, 1999). Thus, they can be simplified to a single surface representing the surface of apposition between the AZ and the PSD. This surface would lie approximately in the middle of the synaptic junction and would adapt to its curvature. The size, position, and shape of this surface will characterize some relevant features of the synapse, including the area of both the AZ and the PSD, the position in space of the synaptic junction and the curvature of the pre- and postsynaptic membranes. For the sake of clarity, we will refer to this surface as the *synaptic apposition surface* (SAS).

## Methods and results

**Figure 1:**
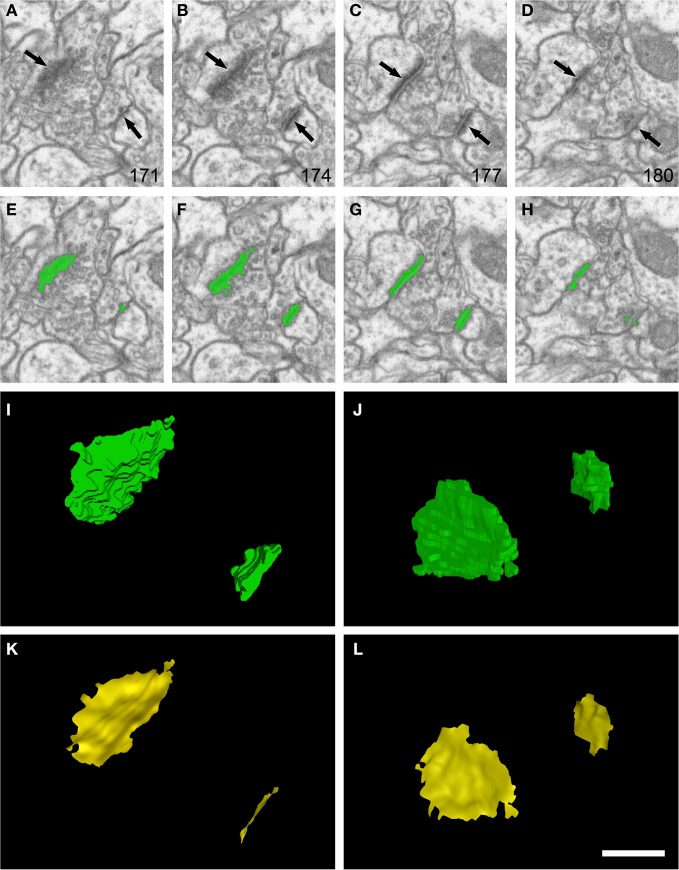
**Example of segmentation and extraction of the synaptic apposition surface from two synaptic junctions. (A–D)**: sections 171, 174, 177, and 180 from a stack of serial images obtained with FIB/SEM from the rat somatosensory cortex. Two synaptic junctions (arrows) can be identified in this series. Images **(E–H)** show the same synaptic junctions (green) after they have been segmented with the software tool ESPINA. Note that segmentation is based on gray-level thresholds, so the pre- and postsynaptic densities are not individually resolvable. **(I)** The synaptic junctions reconstructed in 3D (green). In **(J)** the scene has been rotated 140° about the horizontal axis to show the reconstructed synaptic junctions from a different perspective. **(K)** and **(L)** show the synaptic apposition surfaces (yellow) that have been extracted from the reconstructed synaptic junctions. The original resolution in the x and y axes in **(A–H)** was 3.7 nm/pixel. Resolution in the z axis (equivalent to section thickness) was 20 nm. Scale bar: 500 nm in **(A–H)**; 225 nm in **(I–L)**. See also Video 1 in Supplementary Material.

At present, it is possible to study the ultrastructure of large numbers of synapses within 3D samples of brain tissue. Indeed, using combined focused ion beam milling and scanning electron microscopy (FIB/SEM), it has been shown that virtually all synaptic junctions can be identified regardless of the plane of the section (Merchán-Pérez et al., 2009, 2013; Kreshuk et al., 2011; Blazquez-Llorca et al., 2013). Tissue preparation involves fixation in aldehydes, osmication, *en bloc* staining with uranyl acetate, dehydration, and embedding in Araldite. Stacks of serial images are then obtained by automated FIB/SEM (Merchán-Pérez et al., 2009). Since image segmentation, quantification, and analysis of synaptic junctions in these stacks are all labor-intensive procedures, we have developed ESPINA, a software tool that greatly facilitates and accelerates these processes (Morales et al., 2011a). ESPINA makes use of the fact that presynaptic and postsynaptic densities appear as dark, electron-dense structures under the electron microscope. ESPINA uses a gray-level threshold to extract all the voxels that fit the gray levels of the synaptic junction. The resulting 3D object is irregularly-shaped and flattened, and includes both the pre- and postsynaptic densities and their outer contours (Figures 1A–H). Here we propose a method to extract the SAS, (equivalent to both the AZ and the PSD), from these reconstructed 3D volumes, based on the extraction of interior surfaces from volumetric representations.

The main difficulty when attempting to extract the SAS from actual 3D reconstructions of synaptic junctions resides in the large variability of their size and shape. For example, there are highly tortuous synaptic junctions, and others that have one or several holes (perforated synapses) that also vary in shape, size, and distribution. This variability precludes the use of the techniques currently available, so we have developed a new method to overcome the difficulties associated with SAS extraction. We propose a hybrid solution that obtains the desired result very efficiently by a combination of a deformable template surface and a distance transform method.

### Extraction of internal surfaces

Several different approaches have been proposed to extract internal surfaces from 3D volumes. Skeletonization algorithms (Hisada et al., 2001; Mellado et al., 2007; Cao et al., 2010) are simple to implement but, in our experience, they often require a final post-processing step that involves either a pruning operation or a surface reconstruction process. Thinning methods (Lam et al., 1992) eliminate external voxels from the 3D volume until a planar surface is obtained. Although they are also simple to implement and fast executions can be achieved, thinning methods do not preserve the topology of the object and different results can be obtained depending on the order in which the voxels are removed. Voronoi diagram methods (Okabe et al., 2000) are based on partitioning and triangulating the 3D space. Internal surfaces can be obtained by incrementally removing triangles from the external surface of the volume. Voronoi diagram methods permit topology preservation, but they are computationally expensive in 3D, numerically unstable, and spurious branches must also be pruned at the end of the process. Finally, distance transform methods (Borgefors, 1986) consist of deriving a new volumetric representation from the original reconstructed volumes. Each voxel of the new representation stores its distance to a given reference. In our case, the relevant reference is the external contour of the 3D object (the reconstructed synaptic junction). Distance transforms do not need any post processing step, although the topology is not completely preserved and it may be difficult to locate ridges if the shape is complex. To overcome these drawbacks, we have developed a hybrid approach that is based on a distance transform method and a deformable template surface.

The application of a distance transform to a binary image produces a distance image where each pixel is assigned a distance label. In a 3D object, for each voxel the label stores a value indicating the shortest distance to the external contour. The set of voxels with their corresponding distance labels constitute the distance map (Borgefors, 1986). By making use of the *a priori* knowledge about the shape of the synaptic junction, our problem can be simplified by first placing an initial planar seed or template intersecting the 3D object at the appropriate location, and then deforming this plane according to the shape of the synaptic junction and the distance map values. This approach provides a free-form surface, the SAS, which should fulfill the following criteria: (i) The SAS should lie entirely within the reconstructed synaptic junction, equidistant to its pre- and postsynaptic faces. (ii) The curvature of the SAS should reproduce the curvature of the 3D segmentation of the synaptic junction. (iii) The external perimeter of the SAS should reproduce the external perimeter of the segmentation, as well as the holes that occasionally appear in perforated synaptic junctions.

### Description of the algorithm

**Figure 2:**
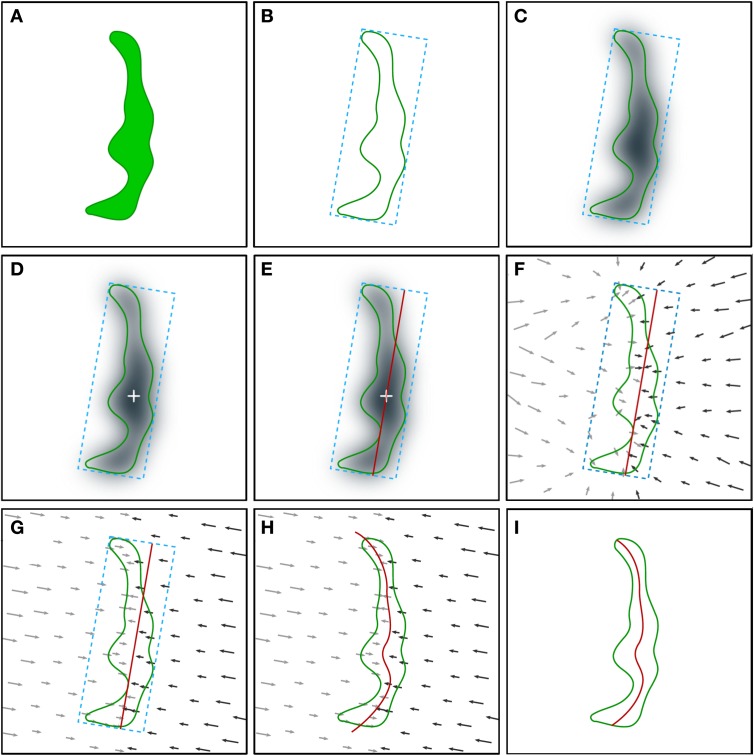
**Schematic illustration of the procedure employed for the extraction of the synaptic apposition surface**. Although the whole process is performed in 3D, it is represented here in 2D for simplicity. **(A)** The starting point is the segmentation of a synaptic junction performed with the software tool ESPINA. The green contour represents the three-dimensionally reconstructed synaptic junction (see also Figure 1 and text for details). **(B)** An oriented bounding box (blue dotted lines) is traced around the reconstructed synaptic junction. **(C)** Computation of the distance map of the segmented synaptic junction. The distance of each voxel to the external contour of the segmentation is measured and each voxel is assigned a distance value. The distance values have been represented by a gray scale. A Gaussian smoothing operation (not shown, see text) is also applied to the distance map. **(D)** Determination of the geometric center (white cross) of the most internal points of the distance map. **(E)** A planar mesh template (red trace) with the same size and orientation as the largest face of the oriented bounding box is generated and placed at the geometric center obtained in the previous step. **(F)** Computation of the gradient of the distance map by substituting scalar distance labels with gradient vectors (arrows). The gradient vectors indicate the deformation that must be applied to the template mesh to reproduce the internal shape of the reconstructed synaptic junction. Gray (lower magnitude) and black (higher magnitude) arrows tend to deform the template mesh in opposite directions. **(G)** Projection of the gradient vectors (arrows) on the normal direction of the template mesh plane. **(H)** The displacement transformation is applied to each vertex of the mesh according to the magnitude of the gradient vectors (arrows). **(I)** The portions of the mesh that protrude outside the segmented synaptic junction are removed by a clipping operation, together with internal holes, if present, in perforated synapses. See also Video 2 in Supplementary Material.

**Figure 3:**
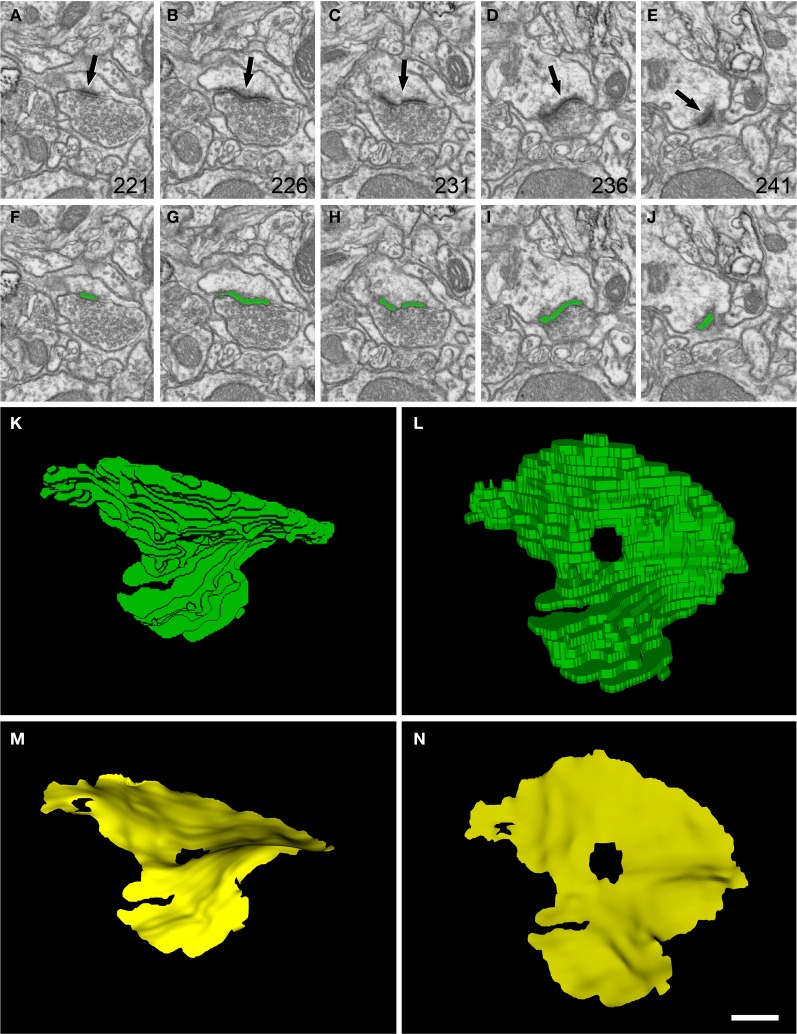
**Segmentation of a perforated synaptic junction and its corresponding SAS. (A–E)**: sections 221, 226, 231, 236, and 241 from a stack of serial images obtained with FIB/SEM from the rat somatosensory cortex. A synaptic junction (arrows) can be identified in this series. Images **(F–J)** show the same synaptic junction (green) after it has been segmented with the software tool ESPINA. The synaptic junction shows a central perforation that is visible in **(C)** and **(H)**. **(K)** The reconstructed synaptic junction is visualized in the same orientation as it was originally sectioned. **(L)** The segmentation has been rotated ~90° about the horizontal axis to clearly show the central perforation. In **(M)** and **(N)** the SAS (yellow) has been extracted from the segmented synaptic junction and is shown with the same orientations as in **(K)** and **(L)**, respectively. The SAS reproduced here shows the curvatures and the irregular contour of the reconstructed synaptic junction. The central perforation is clearly visible, as well as other smaller hole that is not evident in the segmentation. Resolution in the x, y, and z axes was 3.7; 3.7; and 20 nm/pixel, respectively. Scale bar: 500 nm in **(A–J)**; 120 nm in **(K–N)**.

The whole procedure for the extraction of the SAS is performed in the following steps:

- It is necessary to first perform the segmentation of the synaptic junctions that are present in a stack of serial images obtained by FIB/SEM. In the present study this was achieved with ESPINA, a software tool designed for the segmentation of electron microscopy images that uses heuristics based on gray levels and connectivity (Morales et al., 2011a). The result of the segmentation of a synaptic junction is a set of connected voxels that have a gray level over a certain threshold. The contour of these voxels delineates a 3D object that represents the segmented or reconstructed synaptic junction (Figures 1E–H, 2A).
- In order to obtain a spatial reference to locate the deformable template, we have to determine the predominant orientation of the reconstructed synaptic junction. For this purpose, we compute the Oriented Bounding Box (OBB). The OBB is the smallest rectangular box that encloses the segmented synaptic junction (Figure 2B).
- We then obtain the distance map of the segmented synaptic junction by applying the distance transform (Maurer et al., 2003), obtaining a distance value *d*_*i*_ representing the distance between each voxel *i* and the external surface of the reconstructed synaptic junction (Figure 2C). Voxels belonging to the segmented synaptic junction are given a positive distance value while voxels outside the synaptic junction are given a negative distance value. The voxel size of FIB/SEM stacks in the x, y, and z dimensions was either 3.7 × 3.7 × 20 nm or 7.4 × 7.4 × 20 nm. Therefore, any given segmented synaptic junction is not formed by an infinite number of ideal dimensionless points, but is made up by a finite number of polyhedral voxels of these dimensions. Any attempt to extract internal surfaces from these 3D objects would cause curvature artifacts—the curves would appear crooked instead of smooth. To reduce these curvature artifacts we apply a discrete Gaussian smoothing to the distance map (Haralick and Shapiro, 1992):
  $$\begin{array}{l} G(x,y,z)=\frac{1}{\sqrt{2\pi \text{​}\sigma^{2}}}e^{-\frac{x^{2}+y^{2}+z^{2}}{2\sigma^{2}}}\\ \;\sigma =c_{s}\cdot \max(DM) \end{array}$$
  where *c*_*s*_ is an adjustable value between 0 and probit (0.25) = 0.674489 (see below); probit is the quantile function of the Gaussian distribution; and max (*DM*) is the maximum value of the distance map, corresponding to the internal point that is farthest from the 3D object surface.
  Although the value of *c*_*s*_ can be chosen by the user, we have adjusted its default value to *c*_*s*_ = 0.67. The rationale for this choice stems from the properties of the Gaussian smoothing transformation. As an example, we can illustrate the effect of applying the smooth transformation on the distance map of a sphere. In this case, max (*DM*) corresponds to the center of the sphere. When we apply the smoothing algorithm with *c*_*s*_ = 0, no actual smoothing is performed. If we increase *c*_*s*_ values, this results in progressively more intense smoothing until a negative value is obtained for the distance map at the center of the sphere when *c*_*s*_ is larger than 0.674489. The center of the sphere would therefore be labeled as an external point, not belonging to the sphere itself, clearly indicating that the smoothing was too intense. Although higher *c*_*s*_ values would be possible with objects that are flatter than a sphere, we have set *c*_*s*_ = 0.67 as the upper bound, to obtain the maximum smoothing possible without causing undesired artifacts.
- We then compute the best location to place the deformable template within the synaptic junction. To achieve this, we obtain the set of voxels with the highest *d*_*i*_ values in the distance map, corresponding to the most interior points, and we compute the geometric center of this set of points (Figure 2D).
- The deformable template is generated. It is a planar mesh that is oriented along the principal axes of the OBB and intersects the geometric center obtained in the previous step (Figure 2E). The resolution of the mesh (or the number of its vertices) is the same as the number of voxels of the synapse reconstruction that are cut by a plane parallel to the largest face of the OBB.
- The gradient of the distance map gives us a measure of the deformation required to adjust the planar template to the 3D shape of the synaptic junction. We obtain a gradient image (Figure 2F) from the distance map by substituting the distance labels obtained previously with gradient vectors in each voxel *i*:
  $$\nabla d_{i}=\left(\frac{\partial d_{i}}{\partial x},\frac{\partial d_{i}}{\partial y},\frac{\partial d_{i}}{\partial z}\right)$$
- Taking into account the orientation of the synaptic junction, we project the gradient vectors along the normal direction ***n*** of the planar mesh template ∇***d***_*i*_·***n***. As a result, all vectors are now perpendicular to the deformable planar mesh (Figure 2G).
- The deformation is then applied to the template vertices according to the values of the normal vectors calculated in the previous step. The higher the vector value, the more the template mesh vertices must be displaced (Figure 2H). Usually, the positions of the normal vectors do not match the positions of the corresponding template vertices. Therefore, for each vertex to deform, we compute the deformation vector using a cubic interpolation from the nearest vertices. Thus, the positions ***p***_i_ of the template vertices are transformed according to the following expression:
  $$p_{i}(t+1)=p_{i}(t)+\nabla d_{i}\cdot n$$
  This is the only step where several iterations may occur. Since the sequential deformation drives the template vertices to a point of convergence, the stop condition will be reached ideally when the template no longer needs to be deformed. Occasionally the final state is not a unique position but a set of positions between which the template vertices oscillate indefinitely, so we keep a record of the last *s* deformed templates in order to evaluate if the point of convergence has been reached. For each iteration, the mean Euclidean error *e* is computed between the last deformed template and each of the previously recorded *s* templates. If the Euclidean error is less than ε, there are at least two equal states, so the algorithm has converged:
  $$\begin{array}{l} e=\sum_{i=1}^{k}\frac{\left|{\vec{p}}_{i}^{a}-{\vec{p}}_{i}^{b}\right|}{k}\\ \;\varepsilon =c\cdot \vec{\left|n\right|} \end{array}$$
  where *k* is the number of vertices in the grid of the template and ${\overset{\to }{p}}^{a}$, ${\overset{\to }{p}}^{b}$ denote two consecutive states of the template in the iteration process. The parameters chosen represented a trade-off between accuracy and computational resources. We have set *s* = 10 for the number of recorded states and *c* = 0.001 as the threshold factor.
- The portions of the deformed mesh protruding outside the segmented synaptic junction are removed by a clipping operation (Figure 2I). The resulting mesh is the set of facets that constitute the SAS (Figures 1I–L, and Video S2 in the Supplementary Material). In the case of perforated synapses, interior facets that belong to holes are also removed (see Figure 3).

**Figure 4:**
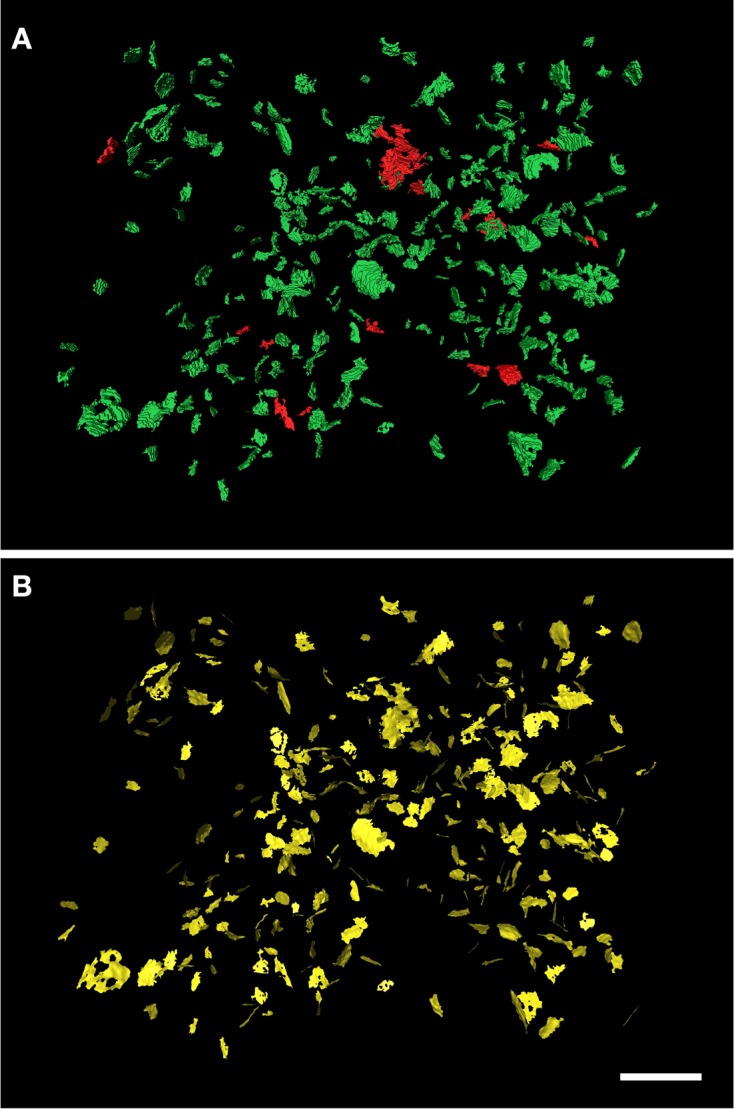
**Extraction of the SAS from a set of 320 synaptic junctions**. The synaptic junctions have been segmented with the software tool ESPINA from a stack of serial sections obtained by FIB/SEM from the rat somatosensory cortex. In **(A)** the three-dimensionally reconstructed segmentations of asymmetric synaptic junctions have been represented in green and those corresponding to symmetric synaptic junctions are depicted in red. **(B)** The extracted SAS have been represented in yellow. Scale bar: 1 μm. See also Video 3 in Supplementary Material.

To test the performance of the algorithm described above, we applied it to two sets of 253 and 320 synaptic junctions. They were segmented with ESPINA from samples obtained by FIB/SEM from the rat somatosensory cortex (Figure 4 and Supplementary Video S3). Over 90% of the synaptic junctions were extracted in less than 0.7 s per junction. The most demanding stage, the computation of the distance map, depends on the number of voxels of the binary image. The maximum size of a single segmentation in our sample was 29,335 voxels, which required a processing time of 38 s. Extraction of all the SAS from the largest set (320 synaptic junctions) took 290 s. Other tests were devised to objectively evaluate the reliability and accuracy of the extraction of the SAS. These tests included the extraction of internal surfaces from 3D objects of known geometric properties (cube, sphere, parallelepiped, and combinations of these objects using constructive solid geometry operations like union, intersection, or difference Boolean operations to obtain more complex shapes with holes). It should be noted that the shape of the SAS will reproduce the shape of the 3D object regardless of whether or not it corresponds to a properly segmented synaptic junction. Therefore, if the segmentation of a given synaptic junction is defective, the SAS will also be defective. This can be avoided during the segmentation step by applying adequate gray-threshold settings and by eliminating noisy pixels in the original stack of images using a Gaussian blur filter, as described elsewhere (Morales et al., 2011a).

The present method has been implemented in C++. We have chosen a set of stable and widely tested free distribution tools to ensure the portability of the code: ITK vs. 3.16 as the image processing library and VTK vs. 5.6 as the mesh manipulation and visualization library. The results have been generated on a PC with Intel Pentium Core I7 920 2.67 GHz CPU and 12 GB RAM memory. Software is available for testing at http://cajalbbp.cesvima.upm.es/sas

### Quantitative geometrical characteristics of the SAS

Several geometrical features of the synaptic junctions are already calculated by ESPINA (Morales et al., 2011a). These include the volume of the synaptic junction, derived from the number of segmented voxels; the dimensions of the bounding box; the binary principal moments; the principal axes of the object; the Feret's diameter, defined as the diameter of the smallest sphere that circumscribes the synaptic junction; and the size of the equivalent ellipsoid, obtained from the principal axes of the synaptic junction. Any of these measurements can be used as a rough estimation of synaptic size, but their interpretation in functionally relevant terms is difficult and all of them lose information about the shape of the synaptic junction. For example, the volume of a given synaptic junction would mainly represent the volume of the PSD. It would be very sensitive to changes of the gray-level threshold used for the segmentation and it is obvious that two synaptic junctions with the same volume may have very different shapes. The equivalent ellipsoid, the dimensions of the bounding box or the principal axes would preserve more information about the shape, but their magnitudes are only roughly related to those of the actual synaptic junction. Finally, the main advantage of the Feret's diameter is its simplicity (Merchán-Pérez et al., 2013), although again it is clear that two objects with the same Feret's diameter may have very different shapes.

**Figure 5:**
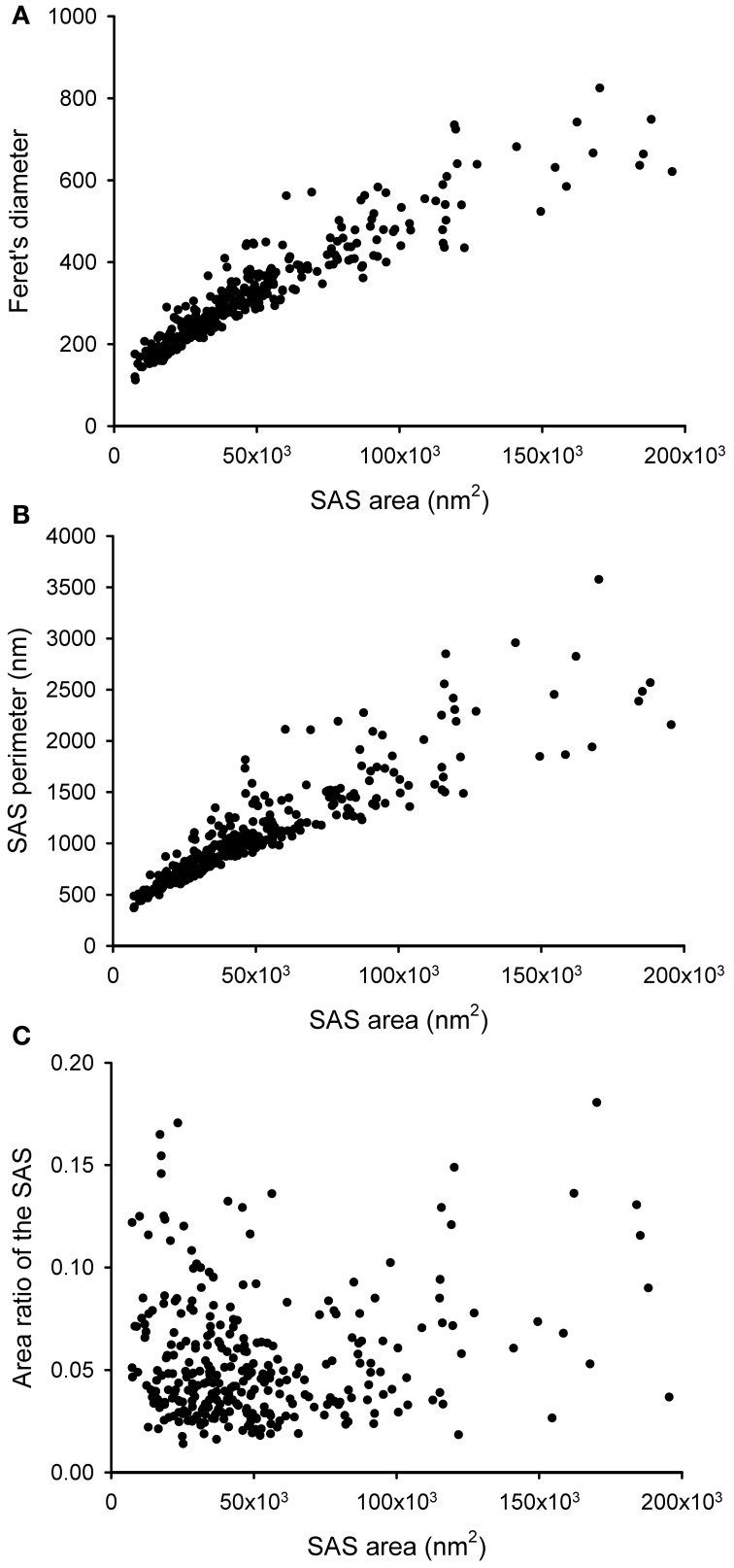
**Some geometrical characteristics of a sample of 320 synaptic junctions that have been reconstructed from a stack of serial sections obtained by FIB/SEM from the rat somatosensory cortex. (A)** Relationship between the area of the SAS and the Feret's diameter (defined as the diameter of the smallest sphere that circumscribes the segmented synaptic junction). Although these two measurements are clearly correlated, two synaptic junctions of a similar Feret's diameter may have a very different SAS area and *vice versa*. Small synapses seem to be more homogeneous than medium and large synaptic junctions. **(B)** Relationship between the SAS area and its perimeter. Again, variability seems to be higher for medium and large synaptic junctions. **(C)** SAS area vs. the area ratio of the SAS. The area ratio of the SAS is one minus the ratio between the projected area of the SAS on the largest face of the OBB and the area of the SAS. The correlation between these two parameters is weak, although there is a tendency for small synaptic junctions to have a flatter profile than medium and large synaptic junctions.

The SAS extracted from a given synaptic junction not only provides qualitative visual information about the shape of the synaptic junction, i.e., whether it is flat, curved or perforated. The SAS can also be quantitatively characterized, providing additional information over and above that which can be obtained from the 3D reconstructed synaptic junctions alone (Figure 5). Since the SAS from every synaptic junction is approximately equivalent to both the AZ and the PSD, its surface area is a relevant measure from a functional point of view. The algorithm presented here calculates the surface area of the SAS, which can be compared with other geometrical features such as the Feret's diameter (Figure 5A). The perimeter is also calculated and, when compared with the surface area, this provides information about the complexity of the SAS contour (Figure 5B). These measurements can be complemented with other measures related to the shape of the SAS.

While the surface area is related to the probability of neurotransmitter release and to the number of postsynaptic receptors, the SAS curvature is related to the shape of the synaptic junction, that is, the shape of the SAS shows whether the synaptic junction is flat, curved, or tortuous. Thus, a measure of the surface curvature allows us to further characterize the shape of the SAS. It is important to find global measures that ensure a robust shape analysis of the SAS features. A simple and intuitive measure involves comparing the surface area of the SAS with its projected area on the largest face of the OBB. This area ratio (Figure 5C) can be defined as follows:

- Area ratio of the SAS: one minus the ratio between the projected area of the SAS on the largest face of the OBB and the area of the SAS. This measure would equal 0 in a perfectly flat SAS, and it would yield progressively higher values as the SAS curvature increases.

$$\text{AR}(\text{SAS})=1-\frac{\text{Area}(\text{ProjectedSAS})}{\text{Area}(\text{SAS})}$$

Other measures of curvature can be extracted locally at each vertex *i* (Zhang and Nagy, 2004):

- Minimum normal curvature at each vertex *i*: κ_1_(*i*)
- Maximum normal curvature at each vertex *i*: κ_2_(*i*)
- Mean normal curvature at each vertex *i*: $H(i)=\frac{\kappa_{1}(i)+\kappa_{2}(i)}{2}$
- Gaussian curvature at each vertex *i*: κ(*i*) = κ_1_(*i*) × κ_2_(*i*)

**Figure 6:**
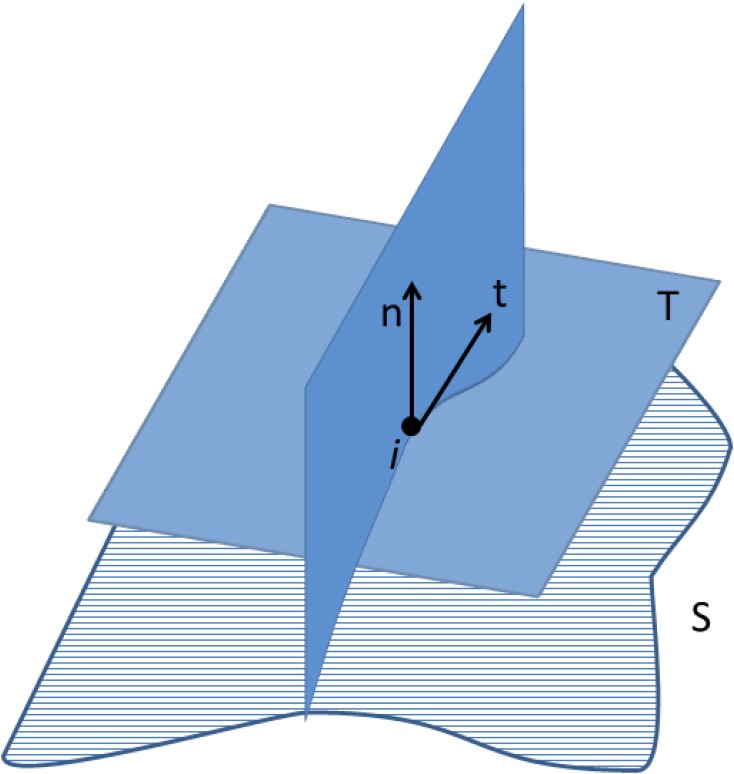
**Curve defined by the intersection of the surface S and the plane determined by the normal vector *n* at vertex *i*, and a vector *t* contained in the tangent plane T**. The minimum and maximum curvatures (κ_1_ and κ_2_, respectively) are determined at each vertex of the surface **S**.

In Euclidean space, κ_1_ and κ_2_ are the maximum and minimum curvatures of the set of curves crossing one vertex *i* of the surface. This set of curves is computed at the intersection of the surface **S** with a plane defined by the surface normal **n** and a vector **t** on the tangent plane **T** (Figure 6). Measures of curvature for the whole set of *n* vertices, based on the local measures described above, have also been implemented in our software, including the following:

- Mean and standard deviation of the minimum and maximum normal curvatures:
  $$\mu \left(\kappa_{j}\right)=\sum_{i=1}^{i=n}\frac{\kappa_{j}(i)}{n}\sigma_{j}=\sqrt[{2}]{\frac{\sum_{i}{\left(\kappa_{j}(i)-\mu (\kappa_{j})\right)}^{2}}{n}},j=1,2$$
- Mean and standard deviation of the mean normal curvature of the SAS:
  $$\mu \left(H\right)=\sum_{i=1}^{i=n}\frac{H(i)}{n}\sigma (H)=\sqrt[{2}]{\frac{\sum_{i}{\left(H(i)-\mu (H)\right)}^{2}}{n}}$$
- Mean and standard deviation of the Gaussian curvature of the SAS:
  $$\mu (\kappa )=\sum_{i=1}^{i=n}\frac{\kappa (i)}{n}\sigma (\kappa )=\sqrt[{2}]{\frac{\sum_{i}{\left(\kappa (i)-\mu (\kappa )\right)}^{2}}{n}}$$
  Where *n* is the total number of vertices.

## Conclusions

Although several geometric characteristics can be extracted from synaptic junctions reconstructed in 3D, they only represent rough estimates of the size and shape of synapses. The SAS, however, is approximately equivalent to the AZ and the PSD. The surface area of the SAS is therefore a biologically relevant measure of synapse geometry since it is related to vesicle release probability at the presynaptic side, and to the number of specific receptors present at the postsynaptic side. Additional information on the shape of the SAS can be obtained by measuring its perimeter, curvature, and tortuosity. We have implemented an algorithm that is capable of extracting the SAS from a 3D volume representing a previously segmented synaptic junction. This algorithm is a hybrid approach that combines a distance transform method and a deformable planar template. The algorithm is fast and does not require user intervention. From a computational point of view, one of its most relevant features is its efficiency and its simplicity, since no complex mathematical background is required. Thus, our algorithm can be applied to the large number of synapses that are currently segmented from stacks of images obtained by three-dimensional electron microscopy techniques such as FIB/SEM. There is considerable interest in determining the geometrical properties of synapses, since changes in the size of the AZ and/or PSD have been reported under a variety of normal, pathological, and experimental conditions and, in general, these changes have been associated with plastic responses and synaptic malfunction. Thus, the combination of FIB/SEM and the algorithm presented here will help to obtain relevant morphological information that will allow the functional characteristics of large numbers of synapses to be correlated with their geometric properties, which is particularly relevant for better understanding the synaptic organization of the brain in both health and disease. Indeed, FIB/SEM technology has already been shown to be useful in the study of alterations of cortical synapses in the brain of patients with Alzheimer's disease (Blazquez-Llorca et al., 2013), as well as in animal models (Alonso-Nanclares et al., 2013).

### Conflict of interest statement

The authors declare that the research was conducted in the absence of any commercial or financial relationships that could be construed as a potential conflict of interest.

## Supplementary material

Video 1 **Extraction of the SAS (yellow) from two segmented synaptic junctions (green)**.

Video 2 **Sequence of events from the segmentation of a synaptic junction (green) to the extraction of the corresponding SAS (red)**.

Video 3 **Extraction of the SAS (yellow) from a set of 320 synaptic junctions**. The synaptic junctions (green) have been segmented with the software tool ESPINA from a stack of serial sections obtained by FIB/SEM from the rat somatosensory cortex.
